# Comparison of the Feasibility and Safety of First- versus Second-Generation AMPLATZER™ Occluders for Left Atrial Appendage Closure

**DOI:** 10.1155/2017/1519362

**Published:** 2017-09-20

**Authors:** Baravan Al-Kassou, Heyder Omran

**Affiliations:** ^1^Medizinische Klinik und Poliklinik II, Universitätsklinikum Bonn, Sigmund-Freud-Str. 25, 53127 Bonn, Germany; ^2^GFO Kliniken Bonn, Robert-Koch-Str. 1, 53115 Bonn, Germany

## Abstract

**Introduction:**

Left atrial appendage closure (LAAC) is considered an alternative to oral anticoagulation therapy in patients with atrial fibrillation (AF). The aim of this study was to compare the safety and efficacy of the first- and second-generation AMPLATZER Devices for LAAC, AMPLATZER Cardiac Plug (ACP) versus AMPLATZER Amulet™.

**Methods:**

Procedural data, such as fluoroscopy time, radiation dose, and contrast-dye, as well as VARC criteria and major adverse events (MAEs) were assessed for both devices. The rate of peridevice leaks was analyzed at echocardiographic follow-up.

**Results:**

A total of 196 patients with AF underwent LAAC with the ACP (*n* = 99) or Amulet device (*n* = 97). The use of Amulet was associated with significantly lower fluoroscopy time (14.8 ± 7.4 min versus 10.6 ± 4.1 min; *p* < 0.001), lower radiation dose (4833 ± 3360 cGy⁎cm^2^ versus 3206 ± 2169 cGy⁎cm^2^; *p* < 0.001), and reduced amount of contrast-dye (150.2 ± 83.9 ml versus 128.8 ± 46.0 ml; *p* = 0.03). Furthermore, LAAC with Amulet devices resulted in lower device-resizing rates (3 versus 16 cases; *p* = 0.001). Peridevice leaks were less frequent in the Amulet group (12 versus 4; *p* = 0.03). MAE occurred in 6 ACP and 4 Amulet patients (*p* = 0.58).

**Conclusions:**

The Amulet device is associated with shorter fluoroscopy times and radiation dosages, reduced use of contrast-dye, lower recapture rates, and less peridevice leaks as compared to the ACP.

## 1. Introduction

The most important complication of patients with nonrheumatic atrial fibrillation (AF) is thromboembolism [1].

Percutaneous closure of the left atrial appendage (LAAC) has been considered a valid alternative to oral anticoagulation therapy for stroke prevention in these patients [2–4].

However, LAAC is still associated with a significant rate of complications, such as pericardial effusion, periprocedural stroke, device embolization, and device thrombosis [5, 6].

Therefore, manufacturers are trying to improve the design of the closure devices to reduce the rate of complications and simplify the implantation procedure.

More recently, St. Jude Medical (Minneapolis, MN, USA) has redesigned the first-generation occlusion device, the AMPLATZER Cardiac Plug (ACP), and introduced the AMPLATZER Amulet [7].

In spite of the same basic structure with a distal lobe and a proximal disc conceived for sealing the body and ostium of the LAA, respectively, the new device has particular features, which are supposed to facilitate the implantation process, improve sealing performance, and further reduce the rate of complications [8].

Smaller studies on the comparison of the older ACP and the new AMPLATZER Amulet device show conflicting data. Abualsaud et al. demonstrated in a study with a total of 59 patients similar procedural and short-term clinical outcome for both devices, whereas peridevice leaks were less frequent with the AMPLATZER Amulet device [9]. Another study with a total of 100 cases failed to show a difference between both devices [10].

The aim of our study was to compare both devices in a larger cohort to

1. evaluate the periprocedural outcome;
2. assess the implantation process, comparing procedure time, radiation dosage, and amount of contrast agent given;
3. evaluate the long term transesophageal echocardiographic outcome.

## 2. Methods

### 2.1. Study Population

A total of 196 consecutive patients with AF and contraindication to effective OAC, who underwent LAAC with the ACP (*n* = 99) and the AMPLATZER Amulet (*n* = 97) between July 2014 and April 2016 at the St. Marien Hospital Bonn, were enrolled in the study.

The procedural performance and angiographic parameters, as well as the clinical outcome and echocardiographic follow-up data of all patients, were collected prospectively and analyzed.

Patient demographics and clinical characteristics, including the CHA_2_DS_2_VASc score and the HASBLED score, were assessed for each patient.

### 2.2. Comparison between the ACP and the AMPLATZER Amulet

Both, first- and second-generation AMPLATZER devices for LAA occlusion are self-expanding devices with a distal lobe and a proximal disc connected by a waist and made from a nitinol mesh. While the distal lobe adapts to the inner LAA wall in the depths, the proximal disk covers the LAA ostium [11].

The second-generation AMPLATZER Amulet has some important modifications, trying to simplify the implantation procedure and to improve the occlusion of the LAA:

The AMPLATZER Amulet is now preloaded inside the delivery system, potentially reducing the risk of air embolization.

The length of the distal lobe is extended in the new devices by 2 to 3 mm, making the distal body more voluminous and better sealing. Furthermore, the increased number of the stabilizing hooks from 6 pairs in the ACP up to 10 pairs in Amulet devices, which also became stiffer now, is intended to reduce device embolization.

Concerning an optimal occlusion of the LAA ostium, the diameter of the proximal disc has been enlarged in the new device, now being 6 to 7 mm greater than the distal lobe diameter. The waist between the proximal disc and distal lobe has also been extended.

In addition, the attaching screw-nut on the disc has been inverted to reduce the occurrence of device thrombosis. Besides, the new AMPLATZER Amulet is available in larger sizes and has a new single-piece cable design for ease of delivery and better control (AMPLATZER Amulet Left Atrial Appendage Occluder Instructions for Use, St. Jude Medical, Minnesota, USA) [7, 8].

### 2.3. Implantation of the Device

The LAAC procedure was performed in all patients under general anesthetics using the ACP (*n* = 99) and the AMPLATZER Amulet (*n* = 96). The implantation was guided by contrast angiography and periprocedural transesophageal echocardiography (TEE) (Vivid E9, GE Medical Systems, Milwaukee, WI, USA), as described previously [5].

The size of the device was determined on the basis of a combination of maximum diameter of the intended landing zone (LZ) on two-dimensional TEE (2DTEE) and contrast angiography as recommended by the manufacturer's instructions for use, as well as visual assessment of the LAA (AMPLATZER Cardiac Plug, AMPLATZER Amulet Left Atrial Appendage Occluder Instructions for Use, St. Jude Medical, Minnesota, USA).

The angiographic assessment of the maximum LZ diameter was performed in the (RAO 30°/10°) cranial and in the caudal view. In the 2DTEE, the maximum diameter at the LZ was obtained in different planes (from 0° to 150° in 30° increments). It was measured from the origin of the left circumflex coronary artery to the roof of the LAA, 1 cm inward from the apex of the ridge separating LAA, and left superior pulmonary vein.

After deployment of the closure device, device stability and position were tested with contrast angiography and TEE. Further details regarding LAAC procedure and special features of the ACP and AMPLATZER Amulet device have been published previously [7, 12].

### 2.4. Angiographic Assessment of the Implantation Procedure

During all LAAC procedures, the time from the beginning of the procedure to the extubation of the patients has been documented. The fluoroscopy time (min) and the radiation dose (cGy*∗*cm^2^), as well as the amount of contrast-dye (ml) given were recorded in all patients using the DAVID® hemodynamic software (Metek, Germany). If the device was recaptured or resized, it was also documented.

### 2.5. Periprocedural Adverse Events

According to the VARC criteria, the collection of the periprocedural adverse events included death, myocardial infarction, stroke, transient ischaemic attack (TIA), systemic embolization, air embolization, device embolization, significant pericardial effusion or cardiac tamponade, and major bleeding [13, 14]. Regarding the safety of the procedure, major adverse events (MAEs) were defined as a composite of periprocedural death, stroke, systemic embolism, and procedure or device-related complications needing major intervention.

### 2.6. Echocardiographic Follow-Up of the Patients

A follow-up TEE was performed 2–6 months after LAAC to assess the closure device stability and to detect potential thrombus and/or peridevice leaks. Therefore, the LAA was systematically scanned in multiple views using color Doppler at the lowest possible Nyquist limit [14]. The observed leaks were classified according to the width of the color jet-flow as previously published: minor leak (jet-flow < 1 mm), moderate leak (jet-flow = 1–3 mm), major leak (jet-flow > 3 mm), or severe leak (multiple jets or free flow) [5, 6].

### 2.7. Statistics

Continuous variables are presented as mean ± standard deviation (SD) and were tested via paired or unpaired Student's *t*-tests. Statistics of categorical variables are presented as absolute numbers and percentages. Fisher exact test was used to assess differences in categorical variables.

Statistical significance was considered as a 2-tailed probability value <0.05. Statistical analyses were performed with SPSS version 23 (IBM Corp., Armonk, NY, USA).

## 3. Results

A total of 196 consecutive patients undergoing LAAC, 99 with ACP from August 2010 to October 2014 and 97 with the Amulet from October 2014 to April 2016, were enrolled in the study. The baseline characteristics of the two patients groups are summarized in Table 1.

There were no significant differences between both groups, except for the medication before LAA occlusion: patients in the ACP group received more often low molecular weight heparin as antithrombotic therapy, whereas patients in the Amulet group were treated more frequently with coumarin derivatives (*n* = 47 for ACP versus *n* = 18 for Amulet, *p* < 0.001; *n* = 20 for ACP versus *n* = 34 for Amulet, *p* = 0.02, resp.).

However, the mean CHA_2_DS_2_-VASc score (4.5 for ACP and Amulet; *p* = 0.9) and the mean HASBLED score (3.4 for ACP versus 3.5 for Amulet; *p* = 0.44) did not differ significantly.

The main indication for LAAC in both groups was previous major bleeding (*n* = 47 (47%) for ACP versus *n* = 50 (52%) for Amulet; *p* = 0.57), followed by previous minor bleeding (*n* = 42 (42%) for ACP versus *n* = 30 (31%) for Amulet; *p* = 0.10) and renal or hepatic disease (*n* = 34 (34%) for ACP versus *n* = 34 (35%) for Amulet; *p* = 0.92)—none presented significant difference (Table 2).

### 3.1. Implantation of the Device and Angiographic Assessment of the Procedure

Table 3 presents the periprocedural data of both groups: the implantation of the ACP device was successful in 96 patients (97%), compared to a successful implantation of the Amulet device in 97 patients (100%) (*p* = 0.08).

The use of the AMPLATZER Amulet was associated with a significant reduction of the fluoroscopy time (14.8 ± 7.4 min for ACP versus 10.6 ± 4.1 min for Amulet; *p* < 0.001) and the radiation dose (4833 ± 3360 cGy*∗*cm^2^ for ACP versus 3206 ± 2169 cGy*∗*cm^2^ for Amulet; *p* < 0.001).

The medium of contrast agent given was also lower in the Amulet group, as compared to the ACP group (150.2 ± 83.9 ml for ACP versus 128.8 ± 46.0 ml for Amulet, *p* = 0.03).

Moreover, recapturing and resizing of the initial selected device size was significantly less necessary in the Amulet group, counting 3 cases (3%) compared to 16 cases (17%; *p* = 0.001) in the ACP group.

However, the total procedural time did not differ significantly in both groups (*p* = 0.09).

### 3.2. Periprocedural Adverse Events

The occurrence of major adverse events was comparable in both groups as shown in Table 4 (*p* = 0.58).

The implantation of the ACP device led to 6 MAEs, including two patients with major bleeding and a patient with in-hospital stroke. The only two procedure-related deaths were reported in the ACP group, one due to device embolization requiring surgery and the other due to periprocedural pulseless electrical activity.

In the Amulet group, a total of 4 MAEs were notified, mainly driven by major bleeding (*n* = 3 (3%)). In one case, a procedure-related cardiac tamponade with need for drainage was observed.

Regarding other adverse events, the ACP and the AMPLATZER Amulet implantation were each associated with 3 vascular complications, such as femoral artery pseudoaneurysm and arteriovenous fistula (*p* = 1).

### 3.3. Transesophageal Echocardiographic Follow-Up

Follow-up TEE was performed in 81 patients (82%) in the ACP group and in 82 patients (85%) in the Amulet group. The mean time duration between the implantation procedure and the first TEE examination was 3.1 ± 6 months for ACP and 2.3 ± 1.5 months for Amulet (*p* = 0.33). As shown in Table 5, none of the patients presented device embolization.

Peridevice leaks were significantly more frequent in patients with ACP devices, counting 12 cases with minor leaks at follow-up TEE compared to 4 minor leaks in the Amulet group (*p* = 0.03). Moreover, the use of Amulet was associated with a reduced rate of device-related thrombus, counting only one Amulet case compared to 4 ACP cases, although not reaching significance (*p* = 0.17).

## 4. Discussion

The major results of the present study with a total of 196 consecutive patients undergoing LAAC with the first and second generation AMPLATZER devices are as follows:

1. The new AMPLATZER Amulet device simplifies the implantation procedure, resulting in significantly shorter fluoroscopy time, less radiation dosage, and a reduced use of contrast-dye.
2. Recapturing and resizing of the occlusion device was significantly lower with the new Amulet device.
3. The use of the AMPLATZER Amulet was associated with a significant reduction of peridevice leaks at follow-up as compared with the ACP.

### 4.1. Procedural Data

Procedural success is generally high with all LAAC devices [15].

Hence our finding of a success rate of 97% for the ACP device and 100% for the Amulet device is in congruence with previous reports [5–7]. Since the rate of success was very high, a statistical difference between both groups is not detectable. This is in agreement with the findings of Gloekler et al., who compared the ACP and Amulet device in a dual-center cohort study of 100 consecutive patients [10].

However, Gloekler et al. did not report on angiographic assessment of the procedure, such as fluoroscopy time and radiation dosage.

We could show that fluoroscopy time was reduced by 28.4% and radiation dosage by 33.6% with the use of the newer device. In concordance to the short fluoroscopy time, the applied amount of contrast agent was 14.2% lower with the Amulet device as compared to the ACP device. This is partly due to the fact that recapturing and resizing of the newer device was only necessary in 3% of cases.

The latter finding is of particular importance, since recapturing and resizing of the device may potentially harm the left atrial appendage.

Since we perform our procedures in general anesthesia, the total duration of the procedure is also influenced by the duration of anesthesia. Hence we did not find a significant difference between both groups.

In addition to the improved implant profile in experienced operators, these findings might also be explained by the modifications of the newer Amulet device improving technical success of the device implantation: the extended body of the device with increased number of the stabilizing hooks may simplify the proper apposition of the device in the LAA and provide more stability.

### 4.2. Periprocedural Adverse Events

In concordance to previous publications, the occurrence of periprocedural major adverse advents was low in both groups [10]. There were 6 (6%) events in the ACP group and 4 (4%) in the Amulet group. Of interest 3 out of the 4 events in the Amulet group were periprocedural major bleedings not directly related to implantation process. We observed only 1 pericardial tamponade in the Amulet group. Despite the trend of lower rates of major complications in the Amulet group, a statistical difference between both groups is not detectable.

### 4.3. Echocardiographic Follow-Up

The TEE follow-up examination showed 4 (5%) thrombi on the device in the ACP group. This is very similar to the finding of Apostolos et al. reporting on 4.4% of thrombi related to the ACP device [5]. Of interest is our observation that only 1 patient in the Amulet group had a thrombus on the device 2 months after implantation. This difference is not statistically significant. However, there is a signal that the larger disc and the embedded screw-nut may potentially reduce thrombus formation on the device.

In concordance with Tzikas et al., we could demonstrate that the use of Amulet device is associated with a significant reduction of peridevice leaks at follow-up TEE examination as compared to the ACP devices [9]. However, the number of peridevice leaks was high in his study, detecting 48% of leaks in the ACP group compared to 8% in the Amulet group. The much larger study on the multicenter experience with the ACP with 1001 patients reported only 11.6% of peridevice leaks [5].

In our series, 15% of the patients with ACP devices and 5% of the patients with Amulet devices presented peridevice leaks, which is in agreement with the larger study. Interestingly all of our peridevice leaks occurring in both groups were minor leaks.

Our finding supports the assumption that the enlarged and more voluminous distal lobe improves sealing performance of the inner LAA in the depths, while the extended diameter of the proximal disc may enhance the covering of the LAA ostium.

## 5. Conclusions

The aims of the development of a second-generation occluder with some important modificants were to improve the implantation process and to further enhance the implantation safety.

The present article shows a better procedural performance of the newer AMPLATZER Amulet device with significantly reduced radiation dosage, fluoroscopy time, and amount of contrast agent given.

Moreover, the reduced occurrence of device-related thrombus and peridevice leaks with the Amulet might be a result of an improved sealing performance due to the extended distal body with enhanced anchoring and the enlarged disc with inverted screw-nut.

## Figures and Tables

**Table 1:** Patient characteristics.

	ACP *n* (%) or value (*n* = 99)	Amulet *n* (%) or value (*n* = 97)	*p*
Age (years)	75 ± 6	74 ± 11	0.36
Age ≥ 75 (years)	56 (57)	53 (55)	0.85
Male	63 (64)	57 (59)	0.43
Body mass index (kg/m^2^)	27.2 ± 5.4	27.8 ± 5.5	0.43
CHA_2_DS_2_VASC score	4.5 ± 1.6	4.5 ± 1.6	0.90
HASBLED score	3.4 ± 0.9	3.5 ± 0.9	0.44
Atrial fibrillation			
Paroxysmal/persistent	41 (41)	53 (55)	0.06
Permanent AF	58 (59)	44 (45)	0.06
*Clinical features*			
Coronary artery disease* *	43 (43)	46 (47)	0.58
Myocardial infarction	19 (19)	16 (16)	0.62
PCI	24 (24)	29 (30)	0.38
CABG	13 (13)	9 (9)	0.40
CMP	12 (12)	13 (13)	0.79
Arterial hypertension	94 (95)	91 (94)	0.73
Diabetes mellitus	26 (26)	34 (35)	0.18
Hyperlipidemia	45 (45)	47 (48)	0.68
Creatinine	1.34 ± 0.8	1.39 ± 0.8	0.68
Quick	87.3 ± 24.5	88.9 ± 26.8	0.67
INR	1.15 ± 0.3	1.17 ± 0.4	0.63
PTT (sec)	27.9 ± 4.7	28.6 ± 6.3	0.44
Nicotine	31 (31)	24 (25)	0.31
*Medication before LAA occlusion*			
Clopidogrel	23 (23)	14 (14)	0.12
Vitamin K antagonist	20 (20)	34 (35)	0.02
New oral anticoagulant drug	19 (19)	29 (30)	0.08
Low molecular weight heparin	47 (47)	18 (19)	<0.001
Beta-blocker	84 (85)	72 (74)	0.06
Statin therapy	43 (43)	42 (43)	0.99
Diuretics	63 (64)	65 (67)	0.62
ACE inhibitor	42 (42)	33 (34)	0.23
*Risk factors for bleeding*			
Previous stroke/TIA	31 (31)	35 (36)	0.48
Prior major bleeding	47 (48)	50 (52)	0.57
Renal disease	31 (31)	33 (34)	0.69
Liver disease	5 (5)	6 (6)	0.73
Labile INR	5 (5)	0 (0)	0.03
Age > 65	91 (92)	84 (87)	0.23

**Table 2:** Indications for left atrial appendage occlusion.

	ACP *n* (%) (*n* = 99)	Amulet *n* (%) (*n* = 97)	*p*
Previous major bleeding	47 (47)	50 (52)	0.57
Intracranial bleeding	15 (15)	18 (19)	0.53
Gastrointestinal bleeding	27 (27)	22 (23)	0.46
Other	5 (5)	9 (9)	0.25
Previous minor bleeding	42 (42)	30 (31)	0.10
Gastrointestinal bleeding	21 (21)	11 (11)	0.06
Hematoma	6 (6)	10 (10)	0.28
Other	15 (15)	9 (9)	0.21
Renal or hepatic disease	34 (34)	34 (35)	0.92
High risk of falls or prior falls	9 (9)	5 (5)	0.29
Physician/patient refusal of oral anticoagulation	0 (0)	2 (2)	0.15

For some patients, more than 1 indication was reported.

**Table 3:** Procedural data.

	ACP *n* (%) or value (*n* = 99)	Amulet *n* (%) or value (*n* = 97)	*p*
Procedural success	96 (97)	97 (100)	0.08
Procedure time (min)	82 ± 28	75.9 ± 23	0.09
Contrast medium (ml)	150.2 ± 83.9	128.8 ± 46.0	0.03
Fluoroscopy time (min)	14.8 ± 7.4	10.6 ± 4.1	<0.001
Radiation dose (cGy*∗*cm^2^)	4833 ± 3360	3206 ± 2169	<0.001
More than 1 device tried	16 (17)	3 (3)	0.001
Larger device finally implanted	5 (5)	1 (1)	0.10
Smaller device finally implanted	11 (11)	2 (2)	0.01
Hospital stay (days)	6.5 ± 5	5.9 ± 4	0.37

**Table 4:** Periprocedural adverse events.

	ACP *n* (%) or value (*n* = 99)	Amulet *n* (%) or value (*n* = 97)	*p*
*Major adverse events*			
Death	2 (2)	0 (0)	0.16
Pulseless electrical activity	Procedure		
Device embolization requiring surgery and leading to bleeding complication	Day 7		
Stroke	1 (1)	0 (0)	0.32
Systemic embolism	0 (0)	0 (0)	1
Myocardial infarction	0 (0)	0 (0)	1
Cardiac tamponade	0 (0)	1 (1)	0.31
Major bleeding	2 (2)	3 (3)	0.64
Intracranial bleeding	1 (1)	0 (0)	0.32
Gastrointestinal	0 (0)	2 (2)	0.15
Epistaxis requiring blood transfusion due to M. Osler	1 (1)	1 (1)	0.99
Device embolization requiring surgery	1 (1)	0 (0)	0.32
Device embolization snared	0 (0)	0 (0)	1
Need for surgery	0 (0)	0 (0)	1
*Total*	*6 (6)*	*4 (4)*	*0.58*
*Other adverse events*			
TIA	0 (0)	0 (0)	1
Air embolism (transient ST elevation and/or chest pain)	0 (0)	0 (0)	1
Vascular complication	3 (3)	3 (3)	0.98
Femoral artery pseudoaneurysm	3 (3)	1 (1)	0.32
Arteriovenous fistula	0 (0)	2 (2)	0.15
*Total*	*3 (3)*	*3 (3)*	*0.98*

**Table 5:** Prevalence and severity of peridevice leaks at transesophageal echocardiographic follow-up.

	ACP *n* (%) (*n* = 81)	Amulet *n* (%) (*n* = 82)	*p*
*Device-related thrombus*	*4 (5)*	*1 (1)*	*0.17*
Device embolization	0 (0)	0 (0)	1
*Peridevice leakage*	*12 (15)*	*4 (5)*	*0.03*
Severe leaks	0 (0)	0 (0)	1
Major leak	0 (0)	0 (0)	1
Moderate leak	0 (0)	0 (0)	1
Minor leak	12 (15)	4 (5)	0.03
